# A Surgical Case

**Published:** 1887-11

**Authors:** Wm. Steinrauf

**Affiliations:** Nokomis, Illinois


### A SURGICAL CASE.

WM. STEINRAUF, M. D., NOKOMIS, ILLINOIS.

I think we would all be the gainers if more interest were taken in surgical matters in our ranks.* Report of surgical cases ought to appear more frequently in our journals than heretofore. There is a belief in some quarters that homœopaths do not practice surgery—in fact, slight it and leave no experience in this branch of the profession. I was in receipt of letters some time ago from prominent men in a city of some seven thousand inhabitants, asking if I knew of a good homœopathist wishing to change his location, with the distinct remark that he ought to be a good obstetrician, but particularly understand surgery. For two years physicians of our school had come and gone in the city referred to, but not staying very long, because, as my correspondent claimed, they could not cope with the allopaths in surgery. Now this should not be. As a matter of course, the homœopathic healing art very often needs no knife and maims and sacrifices no life, where allopaths cut and slash right and left, as with the properly indicated and selected remedy he often succeeds in banishing the threatened disaster and curing with his tinctures, pills, and powders where the "regular" only thinks of scraping, cutting, and sawing.

I have a case in view which has some interesting features, and will relate it.

C. H., æt. nineteen years, was taken, on the first day of February, 1887, with a terrible, painful spot on his left shoulder—deltoid muscle—and a temperature of one hundred and four. As the father is subject to rheumatism, and an older brother has had repeated attacks of acute inflammatory rheumatism, and in absence of any other lesion, we had no hesitancy in pronouncing the trouble rheumatism. For about three weeks we kept the lad on such remedies as Aconite, Rhus tox., Bryonia, Pulsatilla, etc., when the fever abated, temperature went down, and convalescence seemed established. All at once the trouble went to

[* Reports of such surgical cases as this will do Homœopathy no good; the case was badly treated! Giving Aconite, Rhus, Bry., Puls., etc., *in three weeks* was very poor prescribing. Again, the trouble showed itself first in left shoulder, and under this heavy fire, went speedily to the weakest spot—the heel. Under another bombardment with Calc., Phos., Hepar, Sulph., and Silicea, the case went from bad to worse. The strong Sul. acid produced a temporary healing of a sore, which, if "tuberculous," will soon break out again and worse than ever!—EDS.]

the hip and along the leg and finally settled in the heel. Heel began to have a doughy feeling, swell, and become extremely tender to the touch; not much fever. It became evident that the heel needed lancing, which was accordingly done. A thick, scrofulous looking matter escaped after the crucial incision was made. It was clear we had an abscess of tubercular origin to deal with, and that the boy might eventually lose his foot, if not his life. Our patient was all the while taking Calc., Phos., Hepar, Sulph., Silicea, as we saw the necessity for each, besides the very best of eatables, as he could digest them.

With the aid of a probe we could detect necrosed bone, pieces of which began to work out. Daily injections were used of Carbolic acid, *Listerine*, etc., to expedite matters, and cause a more rapid detachment of the decayed portions of the os calcis. But our progress seemed only feeble, weeks and months passing and matters remained in a *statu quo*. Having read in an old number of this journal of the virtue of Sulphuric acid in such cases, we proposed to try it. Five drops of the strong acid were added to four ounces of soft water and then openings injected with this once a day. In a week's time there was a perceptible change. The discharge was growing less, and the parts, heretofore puffy, began to shrink. In three weeks' time the wounds had healed and remained so up to date, now three months. Boy walks around, works, and seems well in every regard.

